# Human microbiome collection

**DOI:** 10.1038/s41598-023-30625-9

**Published:** 2023-03-08

**Authors:** Giulio Maria Pasinetti, Silvia Turroni, Joshua Palmieri, Carlotta De Filippo

**Affiliations:** 1 Department of Neurology, Icahn School of Medicine at Mount Sinai, New York, NY USA; 2 Senior VA Career Scientist, Geriatric Research, Education and Clinical Center, James J. Peters Veterans Affairs Medical Center, Bronx, NY USA; 3 Unit of Microbiome Science and Biotechnology, Department of Pharmacy and Biotechnology, University of Bologna, Via Belmeloro 6, 40126 Bologna, Italy; 4 Institute of Agricultural Biology and Biotechnology (IBBA), National Research Council (CNR), Via Moruzzi 1, 56124 Pisa, Italy

**Keywords:** Molecular biology, Immunology

The human microbiome refers to the complex microbial ecosystems that colonize different niches in our bodies and significantly impact homeostasis and overall health. The most studied is certainly the gut microbiome, but knowledge is also increasing on the oral, cutaneous, vaginal communities, etc.

Research in the human microbiome field is rapidly evolving and fundamentally altering the way we conceptualize and treat disease. Based on this, we believe this Collection addresses the complex role of the human microbiome and its implications for a multitude of diseases and biological dysfunctions.

Despite being confined to the gastrointestinal tract (GI), the impact of the gut microbiome on human physiopathology is proving to be ever more extensive. The gut microbiome itself forms a powerful endocrine organ with a unique metabolome that supplements the host biochemistry beyond what it is designed to provide for itself creating a perfect symbiotic relationship! Microorganisms and their metabolites interact with, and/or cross the protective gut epithelial barrier influencing several physiological systems including the immune system, metabolism, neurological signaling and perhaps most unexpectedly, the brain, giving rise to the gut-brain-axis^1,2^.

Traditionally, the gut microbiome and its impact on human biology have been studied using surveillant and correlative techniques. The composition of the gut microbiome would be determined in the context of a disease state and related to a selection of biological markers such as cytokines, gene expression or a behavioral output. Causality would be established by using germ-free mice or strong antibiotic treatment where the absence of a gut microbiome would create dysbiosis and behavioral or biochemical changes^3,4^. While these seminal studies were absolutely essential to drive our current understanding of the breadth of gut microbiome action through the host physiology, research moving forward must expand and become more sophisticated to address the underlying mechanisms such that the human microbiome can be leveraged upon to develop useful preventive and therapeutic interventions.

Despite the undoubted advantages of animal models, translational research from animals to humans poses a barrier to pushing the boundaries of gut microbiome research which requires creativity and innovation to be overcome by the current and next generation of researchers. Studies investigating the causality of the human microbiome need to use current tools but must also evolve to develop new methods to gain insights into possible causal mechanisms linking the gut microbiome and its metabolome to human disease. Especially for studies of probiotic, prebiotic or symbiotic interventions, translation to human utility requires understanding metabolite production and fate and dissecting common molecular pathways.

Furthermore, to understand the causality of the gut microbiome in human diseases, there is an increasing need for a mechanistic understanding of how the metabolic environment can shape microbial communities, and how metabolites that are produced by the microbial ecosystem can affect the host. While next-generation sequencing provides information on the taxonomic composition of the microbial communities present in a given district and on their metabolic potential, metabolomic analysis represents a direct reading of the function of the system, in particular the intestinal system^5^. Therefore, to elucidate the mechanisms underlying the host-microbe interaction, it is necessary to associate the knowledge of the microbial community with the metabolomic profile, able to represent the phenotype in depth and, therefore, obtain insights into the cellular processes in response to some stimuli or interactions.

This Collection discusses the influence of the various microbiome communities on immune inflammatory signals including cytokines, neuroendocrine hormones, bacterial components, neuroactive molecules, and/or microbial metabolites, among others. Given that the GI microbiome is a critical and often overlooked aspect of innate immunity, the overarching goal of this Collection is to gather recent and current research on the microbiome and its far-reaching impacts on a multitude of pathologies, possibly through immune inflammatory mechanisms.

Together, this Collection reflects an innovative forum of current knowledge for a wide role of the microbiome in human disease. Wong et al., examined the significance of the role the microbiota–gut–brain axis’ bidirectional effects play between the GI and the central nervous system to understand the pathophysiology of psychiatric disorders including autism spectrum disorder (ASD)^6^. They show that the gut microbiome of people with ASD comorbid with functional gastrointestinal disorders (FGID) differed from those with ASD alone by phylogenetically distinct taxa that were of low relative abundance. The study also finds that symptoms of autism, anxiety, and overall psychopathology levels were highest among people with ASD and FGID, and explorative analyses showed that lower alpha diversity and microbiota composition at the phylum level (Firmicutes: Bacteroidetes ratio) correlated with higher anxiety and levels.

Ventin-Holmberg et al., explored the fungal and bacterial gut microbiota in pediatric patients with inflammatory bowel disease (PIBD)^7^. Participants received anti-TNF-α medication IFX with the aim to find microbiota markers for prediction of treatment response. They demonstrated differences in the gut microbiota compositions between response groups to IFX therapy in PIBD, further validated by high predictive power for the bacterial microbiota. Additionally, they found differences in the interkingdom interactions between the response groups. Domènech et al., conducted an explorative comparative analysis of the gut (fecal) and oropharyngeal microbiome between people with obsessive compulsive disorder (OCD) (before and after treatment) and age- and gender-matched healthy controls, via 16S rRNA amplicon sequencing^8^. In summary, their results combined with others indicate that the gut microbiome, through possible increase in inflammation related to overabundance of bacteria in certain genera or decrease in abundance of other genera, might be playing a role in the pathogenesis of OCD. Tentatively, this study lays the groundwork for further explorations to develop possible interventions directed at modulating both the microbiota in OCD patients and its associated mechanisms of effect.

We sincerely hope that future research will be able to cohesively build upon the current foundation of rigorous studies by developing novel and innovative approaches that will advance our understanding of these complex, multi-variable biological mechanisms, particularly in the context of human health.
